# Stakeholder’s perspectives and experiences of patient and public involvement (PPI) in clinical trials in maternal and neonatal healthcare: protocol for a qualitative evidence synthesis

**DOI:** 10.12688/hrbopenres.13731.1

**Published:** 2023-05-12

**Authors:** Kathleen Hannon, Jessica Eustace-Cook, Déirdre Daly, Valerie Smith

**Affiliations:** 1 School of Nursing & Midwifery, Faculty of Health Sciences, Trinity College Dublin, Dublin, D02 T283, Ireland; 2 The Library of Trinity College Dublin, Dublin 2, Dublin, Ireland; 3 Trinity Centre for Maternity Care Research, School of Nursing and Midwifery, Trinity Dublin, Dublin, D02 T283, Ireland

**Keywords:** patient and public involvement, trial methodology, neonatal care, maternal care, clinical trials, GRIPP2

## Abstract

Background: Patient and public involvement (PPI) has the potential to improve the relevance of trial outcomes and improve participant recruitment within clinical trials. However, the literature on PPI approaches, outcomes, and attitudes towards PPI in specific clinical research areas is limited. We are interested to know the current approaches to and views of PPI within maternal and neonatal clinical trials, from the perspective and experience of involved stakeholders.

Methods: A qualitative evidence synthesis (QES) of stakeholders’ perspectives and experiences of PPI will be conducted. Stakeholders will include any individual involved in maternal or neonatal clinical trials with experience of PPI in the area or who expresses their views on PPI. The electronic bibliographic databases CINAHL, MEDLINE, PsycINFO, EMBASE, Web of Science and the Maternity and Infant Care (OVID) will be searched from inception. Qualitative studies, mixed-methods studies where the qualitative data can be extracted independently, and surveys with open-ended qualitative questions, will be included.

Aims: The QES seeks to explore stakeholders’, including PPI contributors, trial participants and guardians, and trial researchers, perspectives and experiences of PPI in maternal and neonatal clinical trials.

Discussion: THE QES will provide an understanding of how PPI is understood, operationalised and experienced by stakeholders in maternal and neonatal clinical trials, with the aim of identifying good practice and areas for improvement.

PROSPERO registration: CRD42023383878 (2 ^nd^ March 2023)

## Introduction

‘Patient and public involvement’ (PPI) is defined as research being conducted *with* and *by* patients or members of the public, as opposed to research being solely conducted *on* these groups ( Dudley *et al.*, 2015b; Staley, 2009). The terminology of ‘PPI’ varies across countries, sometimes being referred to, for example, as ‘consumer engagement’ or ‘public participation’ ( Dudley *et al.*, 2015a; Molloy *et al.*, 2019).

The inclusion of PPI in research is advocated for on pragmatic and ethical grounds ( Bagley *et al.*, 2016); pragmatically in that PPI has the potential to produce more relevant research ( Bagley *et al.*, 2016) and ethically by the belief that those affected by a condition or disease should be involved in research that impacts them ( Crocker *et al.*, 2015). Involving patients, their families, or other members of the public, can help identify critical gaps in the research ( EFCNI, 2017), leading to improvements in what type of research is conducted and how.

While PPI is increasingly encouraged by health research funders ( Dudley *et al.*, 2015b; HRB, 2022), discrepancies can arise between the PPI activities detailed within funding applications and its actual implementation ( Buck *et al.*, 2014). Involvement can be tokenistic, particularly if PPI inclusion is viewed solely as a means to secure grant funding ( Dudley *et al.*, 2015b).

This qualitative evidence synthesis (QES) is being conducted as part of a doctoral study on PPI activity within maternal and neonatal clinical trials. While studies have been conducted on public contributors’ and researchers’ experiences of PPI within clinical trials ( Selman *et al.*, 2021; South *et al.*, 2016), to our knowledge, a QES specifically on stakeholders’ perspectives of PPI within maternal or neonatal clinical trials has not been conducted. The objective of this QES is to synthesise the evidence of stakeholders’ perceptions of PPI in maternal and neonatal clinical trials.

## Protocol

### Inclusion criteria

The SPIDER tool was used to define the inclusion criteria for this QES ( Cooke *et al.*, 2012).

- Sample: Stakeholders in maternal or neonatal clinical trials who have experience of PPI or have expressed their views on PPI. These may include, although not necessarily limited to, trial participants, PPI contributors, and any member of the trial research team (e.g., principal investigators, research midwives/nurses, trial managers, and statisticians).
- Phenomenon of Interest: PPI recruitment and PPI activity at any stage of a maternal or neonatal clinical trial.
- Design: Published and unpublished primary qualitative research of any design.
- Evaluation: Themes representative of stakeholders’ perceptions and experiences of PPI in maternal and neonatal clinical trials.
- Research type: Qualitative research and mixed-methods research where qualitative data can be extracted separately for analysis, quantitative surveys with qualitative open-ended questions and data that can be extracted independently and that have been thematically analysed.

For this QES, clinical trials are, as defined by the World Health Organization (WHO), ‘any research study that prospectively assigns human participants or groups of humans to one or more health-related interventions to evaluate the effects on health outcomes’ ( WHO, 2023). Maternal clinical trials are defined as clinical trials in maternal health, involving trial participants at any stage of pregnancy and up to 6 weeks postpartum. Neonatal trials are defined as clinical trials conducted with neonates during the first 28 days of life. ‘PPI contributor’ refers to a patient or lay individual who is involved at any stage of the trial process, from trial conception to trial dissemination.

### Search strategy

With the assistance of a subject librarian, a search strategy of index terms and keywords was developed from two key concepts; clinical trials in maternal and neonatal health, and patient and public involvement ( Table 1). The electronic bibliographic databases CINAHL, MEDLINE, PsycINFO, EMBASE, Web of Science, and Maternity and Infant Care (OVID) will be searched, with the search terms adapted for each database. Grey literature sources, such as GreyNet, will also be searched.

**Table 1.** Search strategy.

Concept	Index terms and keywords
Concept 1: Patient and public involvement	Index Terms: EMBASE: ‘patient participation’/exp OR ‘stakeholder engagement’/exp OR ‘patient and public involvement’/exp CINAHL: (MH "Consumer Participation") MEDLINE (EBSCO): (MH "Patient Participation") OR (MH "Community Participation+") PsycInfo: DE "Community Involvement" OR DE "Client Participation" Keywords: (public* OR citizen* OR patient* OR user* OR "service user*" OR client* OR "care giver*" OR caregiver* OR carer* OR "lay member*" OR stakeholder* OR consumer* OR representative* OR community) N2 (engag* OR involv* OR participa* OR consult* OR activat* OR partner* OR collab* OR contribut* OR "co-develop*" OR "co-research*" OR "co-lead*" OR "co-produc*" OR "decision-making" OR advisor*" OR "shared leadership") OR TI/AB (ppi* OR "patient involvement*" OR "public involvement*" OR "patient engagement*" OR "public engagement*" OR "patient particip*" OR "citizen science*")
Concept 2: Clinical trials in maternal and neonatal health	Index terms: EMBASE: ('clinical trial'/exp OR 'clinical trial (topic)'/exp) AND ('maternal care'/exp OR ‘obstetrics’/ exp OR 'pregnancy'/exp OR 'pregnant woman'/exp OR 'newborn'/exp OR 'childbirth'/exp OR 'perinatal care'/exp OR 'prenatal care'/exp OR 'puerperium'/exp OR 'infant'/exp) CINAHL: ((MH "Clinical Trials+") AND ((MH "Obstetrics") OR (MH "Pregnancy+") OR (MH "Expectant Mothers") OR (MH "Infant, Newborn+") OR (MH "Childbirth+") OR (MH "Perinatal Care") OR (MH "Prenatal Care") OR (MH "Puerperium") OR (MH "Infant+")) MEDLINE: (MH "Clinical Trials as Topic+") AND ((MH "Maternal Health") OR (MH "Obstetrics") OR (MH "Pregnancy+") OR (MH "Pregnant Women") OR (MH "Infant, Newborn+") OR (MH "Parturition+") OR (MH "Perinatal Care+") OR (MH "Prenatal Care") OR (MH "Postpartum Period+") OR (MH "Infant+")) PsycInfo: DE "Clinical Trials" AND ((((((((DE "Obstetrics") OR (DE "Pregnancy")) OR (DE "Prenatal Care")) OR (DE "Intrapartum Period")) OR (DE "Antepartum Period")) OR (DE "Postnatal Period")) OR (DE "Birth")) OR (DE "Neonatal Period")) Keywords: ("clinical trial* OR trial* OR intervention*) AND (matern* OR obstetric* OR labour OR labor OR pregnant* OR "pregnan* wom*n" OR "expect* mother*" OR birth* OR childbirth* OR "child birth" OR antenatal* OR "ante-natal*" OR antepartum OR ante-partum OR intrapartum OR prenatal* OR "pre-natal*" OR perinatal* OR postpartum* OR puerperium OR parturition OR "post-partum*" OR neonatal* OR neonat* OR newborn* OR "new-born*" OR infant*)

No restrictions on language or date will be applied; that is the databases will be searched from their date of inception to the date of the search. Although studies in any language will be included in the search, only English language studies will be included in the QES. Searching all languages, however, will enable the reviewers to determine how many studies may be excluded based on language, and thus assess the extent of language bias as a potential limitation of the QES.

All retrieved citations will be exported to Endnote (version 20.5) where duplicates will be removed before the citations are transferred to Covidence for screening. Screening will take place in two stages; title and abstract screening, and full text review. First, title and abstracts will be screened against the inclusion criteria by one reviewer (KH). A second reviewer (VS or DD) will screen 20% of the records on title and abstract, and the reviewers’ assessments will be cross-checked for congruency. If reviewer congruency is <80%, based on AMSTAR 2’s recommendation that at least 80% agreement should be achieved ( Shea *et al.*, 2007), all citations will be screened independently by two reviewers. Full text of records will be sourced and each record will be screened for inclusion/exclusion by at least two reviewers working independently. Any disagreements will be resolved through discussion until consensus is achieved. A record will be retained of reasons for exclusion of papers at full text review and reported in the final QES in a PRISMA flow diagram ( Page *et al.*, 2021).

### Data extraction and analysis

One reviewer will extract data from all included studies, which a second reviewer will corroborate. A purposively designed data extraction form will be developed. Data extracted will include study design, aim of study, location and setting, year study was conducted, participant demographics, information on PPI and impact on trial design and outcomes (as described by stakeholders), and findings of experiences and views of PPI.

A thematic analysis will be undertaken, following the approach described by Thomas and Harden (2008) unless another method is more appropriate once the raw data have been assessed. This approach will involve generating codes from study findings (i.e., authors’ interpretations and study participant verbatim quotes) extracted from the included studies. These codes will then be grouped into descriptive categories, followed by the researchers generating new analytical themes from these descriptive categories ( Thomas & Harden, 2008). In order to enhance the trustworthiness of the findings ( Barrett *et al.*, 2020), a reflexive approach will be undertaken throughout this QES. Research diaries will be used to document the process and formation of themes, as well as regular discussions between all three reviewers.

### Methodological quality assessment

The twelve-item assessment criteria checklist by Thomas *et al*. (2003) will be used to assess the methodological quality of the included studies. The tool is widely used for assessing the methodological quality of qualitative research. Two reviewers will independently assess the quality of each included study. Consensus will be reached through discussion or through assessment by a third reviewer, if required. Studies will not be excluded from the review based on judgements of low methodological quality, rather this information will be reported in the findings and highlighted in the discussion, as appropriate.

In addition to a quality assessment, the GRIPP2 short form checklist (GRIPP2-SF) will be utilised to evaluate the reporting of PPI in included studies ( Staniszewska *et al.*, 2017). The GRIPP2 checklists, available in both long- and short-form versions, were developed to strengthen the quality of PPI reporting ( Staniszewska *et al.*, 2017). 

### Assessing the findings

The GRADE-CERQual (Confidence in the Evidence from Review of Qualitative Research) assessment will be used to assess and summarise confidence in the review findings ( Lewin *et al.*, 2018).

### Study status

The protocol has been registered on PROSPERO, and the search strategy developed ( Table 1). The search strategy will be implemented on publication and approval of this protocol.

## Discussion

To our knowledge, this is the first QES on stakeholders’ experiences and perceptions of PPI within clinical trials in neonatal and maternal healthcare.

## Data Availability

Open Science Framework: Stakeholder’s perspectives and experiences of patient and public involvement (PPI) in clinical trials in maternal and neonatal healthcare: protocol for a qualitative evidence synthesis https://doi.org/10.17605/OSF.IO/RD7XH ( Hannon *et al.*, 2023) This project contains the following underlying data: Data Extraction Form.xlsx Open Science Framework: PRISMA-P checklist for ‘Stakeholder’s perspectives and experiences of patient and public involvement (PPI) in clinical trials in maternal and neonatal healthcare: protocol for a qualitative evidence synthesis’. https://doi.org/10.17605/OSF.IO/RD7XH ( Hannon *et al.*, 2023) Data are available under the terms of the Creative Commons Attribution 4.0 International license (CC-BY 4.0).

## Software availability

Endnote and Covidence are proprietary software. Alternative software that can be used includes Zotero for reference management include and Rayyan for conducting literature and systematic reviews.
