# Supplementary material for: Stakeholders’ perspectives and experiences of patient and public involvement (PPI) in clinical trials in maternal and neonatal healthcare: protocol for a qualitative evidence synthesis
Source: HRB Open Res. 2023 Sep 14;6:30. Originally published 2023 May 12. [Version 2] doi: 10.12688/hrbopenres.13731.2 (PMC10570689; doi:10.12688/hrbopenres.13731.2)
Supplement: Peer review history

# Reviewer report: Reviewer response for version 2

**DOI:** 10.21956/hrbopenres.15107.r36152

**Published:** 2023-10-12

**Authors:** Sandy Oliver

**Affiliations:** 1 Social Research Institute, University College London, London, England, UK

The authors have revised their protocol to take into account the diversity of PPI contributors they may encounter when conducting their evidence synthesis.

 Their amendments to the screening procedures (an initial set of titles and abstracts) will allow greater collective reflection on the key concepts being reviewed and greater consistency when interpreting texts.

 Their amendments to the data extraction procedures will allow more sophisticated analysis of PPI contributors and how they have been involved.

Is the study design appropriate for the research question?

Yes

Is the rationale for, and objectives of, the study clearly described?

Yes

Are sufficient details of the methods provided to allow replication by others?

Yes

Are the datasets clearly presented in a useable and accessible format?

Not applicable

Reviewer Expertise:

PPI and research synthesis methodology

I confirm that I have read this submission and believe that I have an appropriate level of expertise to confirm that it is of an acceptable scientific standard.

# Reviewer report: Reviewer response for version 1

**DOI:** 10.21956/hrbopenres.15019.r35025

**Published:** 2023-08-18

**Authors:** Sandy Oliver

**Affiliations:** 1 Social Research Institute, University College London, London, England, UK

This planned qualitative evidence synthesis (QES) will address the important topic of stakeholders’ perspectives and experiences of PPI. The key concepts (stakeholders and PPI) are clearly defined. The methods proposed are well established. I have a couple of suggestions to strengthen the work.

 First, I suggest the authors consider the variety of people who might be part of PPI: individuals who can reflect on their own experience or expectations; and advocates who are well informed by networks of patients and/ or the wider public about a variety of experiences and expectations. I would anticipate the involvement of these different groups suiting different specific purposes and methods for involvement.

 Second, I suggest the authors revisit the procedures for screening search outputs. Their current plans for one reviewer to screen titles and abstracts, followed by a second reviewer screening 20% of titles and abstracts suits the purpose of checking accuracy – the usual purpose for this exercise in effectiveness reviews. However, much of this work involves interpretation. I recommend both reviewers screening independently a small set of titles and abstracts then comparing and discussing any discrepancies so they can understand how they have interpreted the text differently. Taking those different interpretations into account, the definitions and screening guidelines can then be refined to more accurate reflect the concepts of interest in the  eligibility criteria. This can be repeated with another small set of titles and abstracts until the reviewers are satisfied that they have clearly expressed and consistently applied their eligibility criteria.

 At the data extraction stage, I recommend extracting data about the types of individuals involved in the role of patients or the public.

 The protocol states that ‘The GRADE-CERQual (Confidence in the Evidence from Review of Qualitative Research) assessment will be used to assess and summarise confidence in the review findings’. This statement would be clearer if ‘findings’ were replaced by ‘analytical themes’ or ‘synthesis statements’.

 Generally the authors make good use of standardised tools for research synthesis.

 I look forward to reading the resulting synthesis.

Is the study design appropriate for the research question?

Yes

Is the rationale for, and objectives of, the study clearly described?

Yes

Are sufficient details of the methods provided to allow replication by others?

Yes

Are the datasets clearly presented in a useable and accessible format?

Not applicable

Reviewer Expertise:

PPI and research synthesis methodology

I confirm that I have read this submission and believe that I have an appropriate level of expertise to confirm that it is of an acceptable scientific standard, however I have significant reservations, as outlined above.

# Reviewer report: Reviewer response for version 1

**DOI:** 10.21956/hrbopenres.15019.r34365

**Published:** 2023-07-21

**Authors:** julie abayomi

**Affiliations:** 1 Edge Hill University, Ormskirk, England, UK

Thank you for asking me to review this comprehensive and well-written protocol.

 The paper defines and explains PPI clearly, and then goes on to explain the benefits of using this approach in the introduction. It also identifies that a QES regarding stakeholders' perspectives of PPI in a maternal or neonatal setting appears not to exist, and so identifies a gap in knowledge.

 For the QES, the SPIDER tool will be employed to define inclusion criteria, ensuring rigour within the process. Furthermore, the search will be supported by a subject librarian, with relevant expertise. The search terms and databases suggested are all highly relevant.

 The authors explain how screening will be conducted and intend to use a PRISMA flow diagram in their report to show this. Extracted data will be analysed thematically, using an agreed approach (Thomas and Harden 2008).

 The quality of the research will be ensured by using an assessment checklist (Thomas et al 2003) and by referring to GRIPP2 when evaluating the reporting of PPI included in studies - this also ensures rigour and structure to the process. Another tool, GRADE-CERQual will be used to assess and summarise confidence, so rigorous methods are employed throughout the whole study.

 Using Endnote, Covidence and Rayyan will all help to streamline the process.

 My only suggestion is that the team may find it helpful to consider/refer to UK Health Research Authority (HRA) Guidelines regarding PPI ( https://www.hra.nhs.uk/planning-and-improving-research/best-practice/public-involvement/ ). This covers the 4 principles for meaningful PPI in health research.

Is the study design appropriate for the research question?

Yes

Is the rationale for, and objectives of, the study clearly described?

Yes

Are sufficient details of the methods provided to allow replication by others?

Yes

Are the datasets clearly presented in a useable and accessible format?

Not applicable

Reviewer Expertise:

Maternal nutrition and health, including PPI with service users and midwives.

I confirm that I have read this submission and believe that I have an appropriate level of expertise to confirm that it is of an acceptable scientific standard.
